# Valgus knee deserves personalized total knee arthroplasty

**DOI:** 10.1530/EOR-2025-0046

**Published:** 2026-01-09

**Authors:** Gautier Beckers, Marc-Olivier Kiss, Vincent Massé, Pascal-André Vendittoli

**Affiliations:** ^1^ Department of Orthopaedic Surgery, Klinikum Großhadern, Ludwig-Maximilians-University of Munich, Munich, Germany; ^2^ Surgery Department, Hospital Maisonneuve-Rosemont, Montreal University, Montreal, Quebec, Canada; ^3^ Personalized Arthroplasty Society, Atlanta, Georgia, USA; ^4^ Clinique Orthopédique Duval, Laval, Quebec, Canada

**Keywords:** valgus, knee, total knee arthroplasty, restricted kinematic alignment, personalized arthroplasty

## Abstract

- Valgus accounts for 18.5% of patients undergoing a total knee arthroplasty (TKA). Following a mechanical alignment (MA) surgical technique, these patients have historically been more challenging than their varus counterparts.
- In valgus knees, conventional MA-TKA frequently distalizes and posteriorizes the lateral femoral condyle, increasing lateral patellar retinaculum tension and flexion space imbalance and instability.
- Personalized arthroplasty is gaining popularity for varus knees, but its value remains debated for valgus knees. This reluctance stems from outdated misconceptions about valgus knee anatomy and biomechanics and limited awareness of advancements in implant survivorship and outcomes.
- Patients with valgus HKA may present with various knee laxities. While medial collateral ligament (MCL) pseudo-laxity and generalized hyperlaxity are easy to manage, true MCL elongation requires careful evaluation and may necessitate surgical modifications.
- A surgical approach favoring patellar tracking and avoiding increasing medial compartment gaps is of paramount importance. Joint laxity assessment should guide surgical decisions, from tibial undercutting for mild laxity to soft tissue releases or constrained implants for severe instability.
- In the presence of a pathological patellofemoral joint, the surgical technique should be adapted with trochlear position/orientation modifications, patellar resurfacing medializing the implant, lateral retinacular release, or a tibial tuberosity osteotomy.
- Long-term studies show high patient satisfaction with restricted kinematic alignment, TKA in valgus knees, with outcomes comparable to varus knees.

## Introduction

Total knee arthroplasty (TKA) has significantly evolved over the years. Owing to the implementation of enhanced recovery after surgery (ERAS) protocols, modernized implant designs, and personalized alignment strategies, the historical dissatisfaction rate of 20% has significantly improved (1, 2, 3). A recent long-term follow-up study on restricted kinematic alignment (rKA) TKA reported that 95% of patients were satisfied or very satisfied with their outcomes (4). While the clinical benefits of personalized surgical approaches for varus knees are increasingly recognized, skepticism remains regarding their benefits for valgus knees (5). This belief is rooted in outdated misconceptions about valgus knee anatomy and biomechanics and a lack of awareness of recent improvements in implant survivorship and outcomes data. This paper aims to review several key features of valgus knee anatomy and biomechanics, recent advancements in implant design, and new data on the outcomes of personalized arthroplasty in valgus knees.

## Valgus knee anatomy

Very few patients have a neutral lower limb hip-knee-ankle (HKA) angle: only 4% of tibias and 5% of femurs have a neutral angle (0°), with just 0.1% having both neutral (6). In a large cohort of osteoarthritic patients, 18.5% (*n* = 2,261/11,991) were found to have a valgus HKA (7). Valgus alignment may result from the joint surface orientation of the femur and/or tibia, extra-articular diaphyseal angulation, asymmetric joint gaps, or a combination of these factors. Moreover, valgus lower limb HKA has been linked to other specific anatomical variations, such as increased collateral ligament laxity, trochlear dysplasia, patella alta, femoral neck anteversion and coxa valga, external tibial torsion, pes planus, and increased hindfoot valgus, among others (8, 9, 10, 11, 12, 13). These anatomical variations should be considered during a personalized TKA procedure.

### Femur anatomy (distal)

Compared to varus knees, valgus knees have a more valgus-oriented distal femoral joint line (6). A study by Alghamdi *et al.* demonstrated that in patients without tibia valga, the mean lateral distal femoral angle (LDFA) was 88.8° in varus knees versus 83.3° in valgus knees (14). One of the persistent myths regarding valgus knees is the belief that valgus femoral orientation originates from lateral condyle hypoplasia (15). Indeed, an analysis of 6,829 knees measuring the difference in radii between the medial and lateral condyles along the femur’s flexion axis demonstrated that the medial condyle is, on average, 1.4 mm smaller than the lateral condyle (16). Similarly, Howell *et al.* found that the lateral femoral condyle in valgus knees is 0.2 mm larger than the medial condyle (17). The valgus joint surface orientation originates from metaphyseal angulation. The traditional approach of under-resecting the distal and posterior lateral condyle by adding varus and externally rotating the femoral component to compensate for the so-called ‘hypoplasia of the lateral femoral condyle’ is, therefore, a flawed concept (3).

### Trochlea anatomy and the patellofemoral joint

Valgus knee alignment is recognized as a risk factor for patellar instability (12). Several anatomical features of the femur, including trochlear groove shape, orientation, and depth (trochlear dysplasia), can contribute to patellar maltracking (12, 18). A flatter patellar anatomy with a wider lateral facet and a larger Wiberg angle is also associated with patellofemoral instability (19). Although the proportion of patients presenting with preoperative patellar tilt, subluxation, lateral retinacular contracture, or a history of patellar instability is relatively low (category 4, Personalized Arthroplasty Society (PAS) classification (20)), performing TKA in these cases requires technical adjustments and an adapted approach to optimize patellofemoral function postoperatively. Replicating the patient’s anatomy with kinematic alignment (KA) may inadvertently reproduce the pathological patellofemoral joint (21). Several factors, such as patellar height (patella alta), Q-angle, increased femoral anteversion, and increased external tibial rotation, play critical roles in guiding patellar movement within the knee (12, 13). Careful attention to these details and addressing them as needed during TKA can help reduce the risk of patellofemoral complications and improve surgical outcomes for patients with valgus anatomy. Recently, Talbot *et al.* concluded that quadriceps tendon malalignment was associated with lateral facet patellar osteoarthritis, patellofemoral joint pathology, and an increased risk of poor TKA outcomes (22). However, another study of 313 KA-TKA cases did not observe such a correlation (23).

### Tibia anatomy

In patients with valgus HKA, the tibial joint surface is less varus than in varus HKA knees, and in some cases, the tibial mechanical axis is neutral or even in valgus (6, 14). A study by Alghamdi *et al.* found that in knees without tibia valga deformity, the mean medial proximal tibial angle was 86.3° in varus knees versus 90.8° in valgus knees (14). Furthermore, they observed tibia valga (extra-articular tibial angulation) in 53% of valgus knees scheduled for TKA, with a mean diaphyseal deformity of 5° (ranging from 3 to 13°). In contrast, diaphyseal angulation was present in only 1% of the varus group (14). When performing mechanical alignment (MA) TKA, recognizing this extra-articular anatomy on preoperative long-leg radiographs is crucial. Compensating for the angulation with intra-articular bone cuts can create soft tissue imbalances, potentially necessitating soft tissue releases or constrained implants (11, 24).

### Femorotibial joint surfaces orientation

Similar to varus knees, MacDessi *et al.* found that in valgus arthritic knees, the most common joint line orientation was apex distal (66.4%), followed by ‘neutral’ (31.9%), and ‘apex proximal’ (1.7%) (25).

### Soft tissues

**Figure 1:**
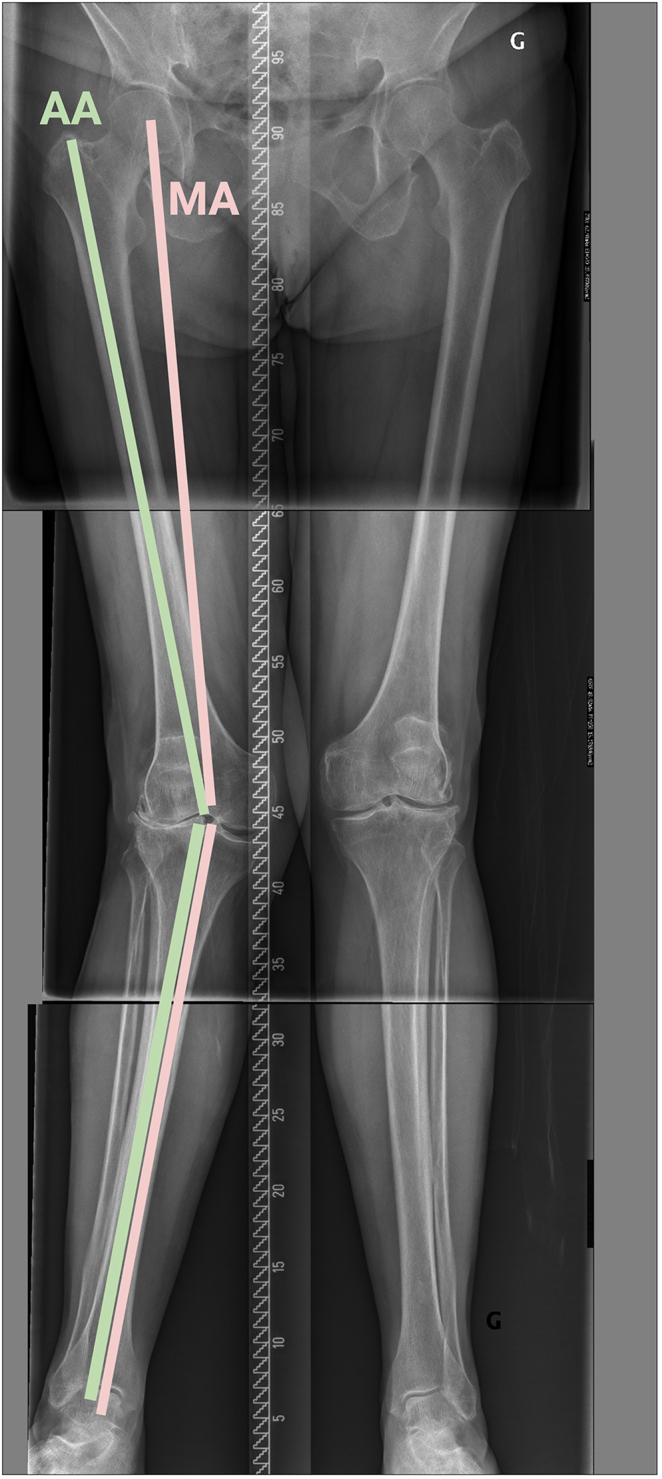
This figure shows the difference between the anatomical and mechanical HKAs. In this example, the angle between the anatomical femoral and tibial axes corresponds to a HKA of 23° valgus, while the mechanical HKA is 17°. Both measurements include the joint wear and opening, while the arithmetic HKA, excluding those, results in a valgus of 6° (distal femur in 9° valgus and proximal tibia in 3° varus). AA, anatomical axis; MA, mechanical axis.

Clinically, patients with valgus HKA are recognized as having physiologically looser knees. With pathological kinematics, subsequent medial collateral ligament (MCL) laxity and/or insufficiency has been reported, leading to a classification based on MCL laxity (26). However, a recent study did not find radiographic evidence of MCL elongation in patients with valgus knees with anatomic femorotibial angles ranging from 11 to 23° (27). These results should be interpreted cautiously, as the reported angles do not represent the mechanical HKA and include cartilage/bone wear and joint opening. In addition, only 26 patients in the study were in valgus (Fig. 1).

During clinical examination, collateral ligament pseudolaxity, defined as apparent laxity caused by intra-articular changes such as bone and cartilage loss that result in increased apparent joint mobility, should not be mistaken for the patient’s native soft tissue laxity (28). In addition, the combination of lateral soft tissue contracture (primarily involving the capsule and popliteus) and slight knee flexion can create a false impression of MCL elongation on standard anteroposterior radiographs (27). Therefore, a thorough assessment of medial laxity is essential both preoperatively and intraoperatively. It is important to distinguish between MCL elongation, generalized ligament hyperlaxity, and pseudolaxity. While MCL pseudolaxity and generalized hyperlaxity are relatively straightforward to identify, true MCL elongation requires careful evaluation and may necessitate modifications in surgical technique or compromises. Strategies for managing these conditions will be discussed later in the text.

### Other anatomical contributing factors

When addressing valgus osteoarthritic knee disease, it is essential to consider the anatomy and function of adjacent joints, as they influence knee alignment and biomechanics.

Proximal femoral variants, such as coxa valga, shift the mechanical axis laterally in the knee, increasing pressure on the lateral knee compartment and favoring genu valgum (8). Patients with lateral knee osteoarthritis tend to have a wider pelvis, while those with medial knee osteoarthritis typically have a higher femoral offset. When performing TKA, surgeons should consider proximal femoral anatomical modifications resulting from a nonanatomical hip replacement. In addition, long-bone extra-articular deformities should be ruled out preoperatively using long-leg radiographs, as such deformities may require a specific surgical approach (11).

Foot anatomy also impacts lower limb alignment. Traditionally, lower limb alignment assessments exclude the hindfoot axis, providing an incomplete representation. Recently, authors have suggested including the calcaneus as the distal landmark for lower limb alignment assessment (29, 30, 31, 32).

Approximately 20% of adults have a flatfoot deformity (pes planus), which is closely associated with genu valgum and may contribute to the progression of knee osteoarthritis (9, 10). Moreover, hindfoot deformities can cause internal tibial rotation, exacerbating valgus knee alignment (9, 10). Therefore, hindfoot alignment should be thoroughly evaluated and, in some cases, addressed concomitantly to optimize TKA outcomes.

Finally, the contralateral lower limb’s anatomy and kinematics may also play a role. Recent data indicate that only 26% of patients exhibit identical coronal functional knee phenotypes in both knees (33). Unilateral valgus alignment may negatively impact gait, and some have suggested that anatomical correction should be considered in such cases (avoiding KA) (11, 34). Furthermore, unilateral severe valgus deformity increases the adduction moment on the contralateral limb, potentially leading to a varus deformity, as seen in some windswept deformities (11, 35). Windswept deformity is observed in 0.8% of patients undergoing TKA. Howell *et al.* demonstrated that both knees can be successfully managed using a personalized alignment strategy, resulting in similar lower limb alignments and outcome scores compared to paired varus and valgus cases (36). Finally, delaying treatment of a severe contralateral varus knee may negatively impact the clinical outcomes of the valgus-side TKA.

Dynamic alignment: we define the knee as varus or valgus based on a static measure of the HKA angle, and this measurement often influences treatment approaches and alignment philosophies. The dynamic HKA measures the knee’s alignment throughout the entire gait cycle by tracking changes in the HKA during movement, serving as a strong predictor of the dynamic load on the knee. Studies have shown that the static HKA angle differs from the dynamic HKA (37, 38), with no correlation between the two for valgus knees, and only a low to moderate correlation in varus knees (38). Furthermore, 22% of the static valgus switched into varus, particularly during the stance phase. This variability limit brings up the complexity of lower limb kinematics and the limited value of static long-leg radiographs as a knee arthroplasty determinant. Given the above and the fact that the absolute variation of the dynamic HKA during gait was 10.9° (range: 2.4–28.3°), aiming for a static neutral alignment with MA appears to be of limited value to predict implant survivorship and clinical results (38, 39).

## Impacts of mechanical alignment on valgus knee anatomy

Because very few patients have a native neutral alignment, the MA technique significantly alters the patient’s native joint line orientation, femoral flexion axis, soft tissue laxities, overall knee kinematics, and patellar tracking (3, 6, 40, 41).

Extension space is influenced by the orientation of the distal femoral and proximal tibial cuts. Since the femoral joint surface in valgus knees is more valgus-oriented than in varus knees (6), performing MA-TKA with a measured resection based on the medial condyle results in an average 4 mm distalization of the lateral condyle in valgus knees. This lateral condyle distalization increases tension on the lateral patellar retinaculum and may contribute to several postoperative patellofemoral complications, including excessive pressure, tilt, subluxation, wear, and pain (42). In addition, valgus knees’ tibial joint surface is less varus-oriented than that in varus knees (6, 14). In an MA-TKA simulation of 270 valgus knees, a neutral tibial cut created an extension space imbalance of ≥3 mm in 54% of cases (40).

**Figure 2:**
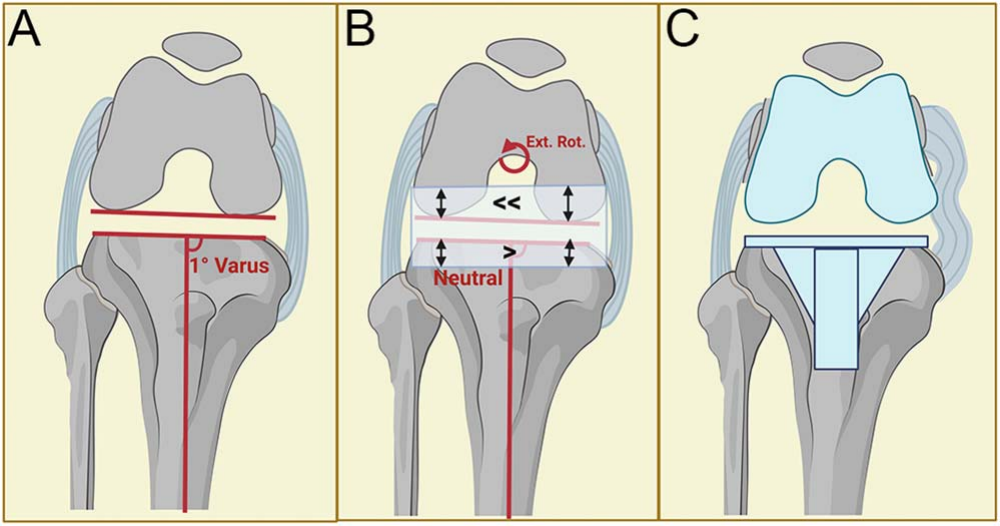
Flexion space with MA. (A) Illustration of a valgus knee at 90 degrees of flexion. Symmetric flexion space before bone cuts. (B) With MA, the proximal tibial cut resects slightly more bone laterally than medially to achieve a neutral cut. As per the MA technique, a systematic 3 or 5° external rotation to the posterior condyles results in greater bone removal from the medial posterior condyle than the lateral side. (C) Unmatched tibial and femoral bone resections create ligamentous imbalance and a looser medial compartment. Ext. Rot., external rotation.

**Figure 3:**
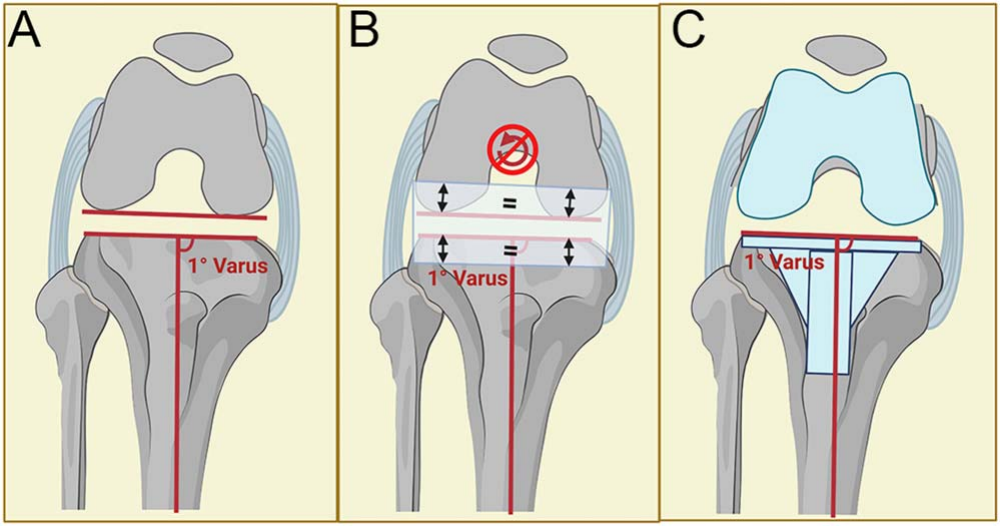
Flexion space with rKA. (A) Illustration of a valgus knee in 90 degrees of flexion. Symmetric flexion space before bone cuts. (B) rKA protocol resurfaces the knee, where equal bone thickness is removed medially and laterally from the proximal tibia and posterior femur (no external rotation). (C) The TKA ligaments are balanced, and the preoperative native laxities are reproduced.

Flexion space is determined by the orientation of the posterior femoral and proximal tibial cuts. Performing MA neutralizes the native tibial varus joint surface orientation (mean of 3°) (42, 43). Insall suggested applying external rotation to the posterior femoral condyles to compensate for this alteration. While this strategy may benefit patients whose anatomy closely aligns with the mean value (3° of tibial varus), it is less suitable for valgus knees. Given that the tibial joint surface in valgus knees is closer to neutral or even in valgus, applying 3° of external rotation to the posterior condyles can create a flexion space imbalance of >3 mm in 23% of valgus knees (Figs 2 and 3) (40). If external rotation is considered, it should be performed about a posteromedial pivot. Using a central or lateral pivot in valgus knees, where the tibia is frequently neutral or valgus, risks opening the medial flexion gap and creating mid-flexion instability (3, 14). Furthermore, it has been common practice in the MA technique to further increase external rotation for valgus knees to ‘correct the hypoplastic lateral condyle’. However, this has resulted in an even greater imbalance in the flexion space.

Because MA alters both extension and flexion spaces in valgus knees to a greater extent than in varus knees (40), soft tissue releases are frequently required to achieve balance, making valgus knees more challenging to manage than varus knees. However, soft tissue releases yield inconsistent outcomes and can introduce new imbalances (44, 45), potentially leading to iatrogenic knee instability and significantly increasing the likelihood of requiring a more constrained implant (20, 27). Understanding the impact of MA on valgus knees helps explain why these knees have historically underperformed compared to neutral and varus knees.

## Implant design

TKA implants play a crucial role in the success of surgical treatment, and their design has evolved significantly due to advancements in manufacturing and a better understanding of knee anatomy. While off-the-shelf femoral component sizes and geometries have expanded, only patient-specific (custom) implants can address each anatomical factor individually, minimizing compromises (46).

The femoral morphology of valgus knees differs from that of varus knees, but there is also considerable variation within the valgus group. Anatomical variations in the distal femur include multiple parameters, such as mediolateral size (wide or narrow), the trapezoidal shape of the distal femur (47), condylar anatomy (curvature radii), and trochlear shape and angle. Given its significance and the unique considerations in valgus knees, the following sections will focus on the latter.

### Implant trochlea’s geometry and orientation

While modern TKA designs feature more anatomically shaped trochlear geometries compared to earlier models (48), a recent study analyzing the native trochlear angles of 4,116 knees using CT imaging – along with the design of 89 currently available total knee prostheses – found a significant mismatch between native and prosthetic trochlear angles (49). The median prosthetic trochlear angle was 6.2°, whereas the native trochlear angle averaged 1.6° (valgus), with a wide range from −23.8° (varus) to 30.3° (valgus). Approximately 60% of native trochlear angles were in valgus, while 40% were in varus (49). A raised lateral trochlea ridge, a feature designed to help prevent patellar tilt and subluxation, can benefit MA when femoral external rotation is applied. However, implantation in neutral rotation may increase lateral retinacular tension and contribute to patellar tilt (50).

When performing personalized TKA for valgus knees, some implants designed for MA have a more favorable trochlear design than others. Implants with a wider trochlea and low lateral ridge help optimize patellar tracking and reduce the risk of patellar malalignment or instability, and are therefore favored by the authors.

To address high patient anatomy variability, two options were proposed:

- Implants with a wider trochlear groove.
- Implant customization to better match patients’ anatomy.

Some manufacturers have modified their implant designs by widening the trochlear groove to improve patellar capture and by enhancing anterolateral femoral coverage to optimize patellofemoral kinematics in KA-TKAs (51). Sappey-Marinier *et al.* (51) demonstrated that KA-TKA using these modified implants resulted in a postoperative trochlear groove positioned lateral to the quadriceps line of force in 100% of cases, effectively accommodating the Q-angle. In contrast, KA-TKA with standard implants achieved this alignment in only 69% of cases.

**Figure 4:**
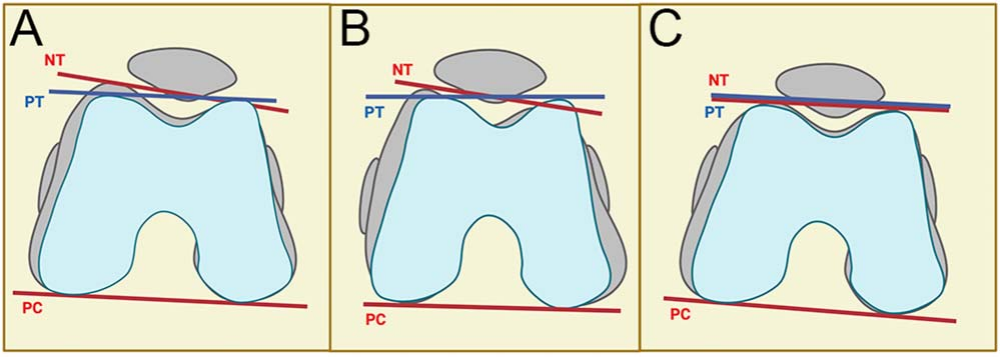
Relation between the trochlea plane versus posterior condyles angle. (A and B) Illustration of the distal femur in flexion showing the relationship between the posterior condyles, the prosthetic, and the native trochlea orientations. Because of the considerable inter-individual variation, resurfacing the posterior condyles using an off-the-shelf implant fails to reproduce the native trochlea. (C) Sometimes, the patient’s anatomy matches the implant’s design and replicates both the anterior and posterior joint surfaces’ orientations. NT, native trochlea; PT, prosthetic trochlea; PC, posterior condyles.

Some researchers suggest that patient-specific implants may offer superior outcomes by replicating the native trochlear alignment, shape, thickness, and relationship to the posterior condyles (52). A key advantage of custom implants is their ability to decouple the patellofemoral and tibiofemoral compartments, preserving the native position and orientation of the trochlear groove relative to the femoral condyles (Fig. 4). Early results indicate that custom implants result in less postoperative patellar tilt than off-the-shelf implants, improving patellar tracking and overall knee function (52). However, the limitations of such customization remain undefined, particularly in avoiding the replication of pathological patellofemoral joint anatomies.

When performing personalized TKA for valgus knees, certain implants originally designed for MA may feature more favorable trochlear designs than others. Implants with a wider trochlea and lower lateral ridge help optimize patellar tracking, reducing the risk of patellar malalignment or instability. For this reason, such designs are favored by the authors.

Tibial baseplate and bearing design should also be considered when performing TKA in valgus knees. Anatomically shaped tibial baseplates optimize bone coverage and help prevent tibial tubercle medialization, which can occur with the external rotation of symmetric baseplates (53).

A medially constrained bearing combined with a flat lateral surface may offer additional benefits by inducing internal tibial rotation, thereby moving the tibial tubercle anteriorly. This adjustment can improve patellar tracking and reduce patellofemoral pressure (54, 55). However, to the best of the authors’ knowledge, such bearing designs are not currently available with custom implants.

## Personalized surgical technique for the valgus knees

### Surgical approach

Using the subvastus or midvastus approach, as the vastus medialis muscle helps stabilize the patella, may improve its tracking (56). In addition, performing a medial parapatellar approach associated with extended lateral retinaculum release, performed in cases of severe lateral retinaculum contracture, could jeopardize the blood supply of the patella, leading to avascular necrosis. During the approach, it is essential to avoid releasing the deep MCL from the medial tibial plateau. Any deep MCL release will create or increase mediolateral imbalance by enlarging the medial gap. In the authors’ experience, most of the procedure can be performed without using a medial retractor, and every precaution should be taken to avoid stretching the MCL. We reserve a lateral surgical approach for cases involving a subluxed patella combined with severe lateral retinacular contracture. The decision is made when the patella remains laterally subluxed and cannot be reduced medially with the knee in full extension.

### Joint laxity assessment

**Figure 5:**
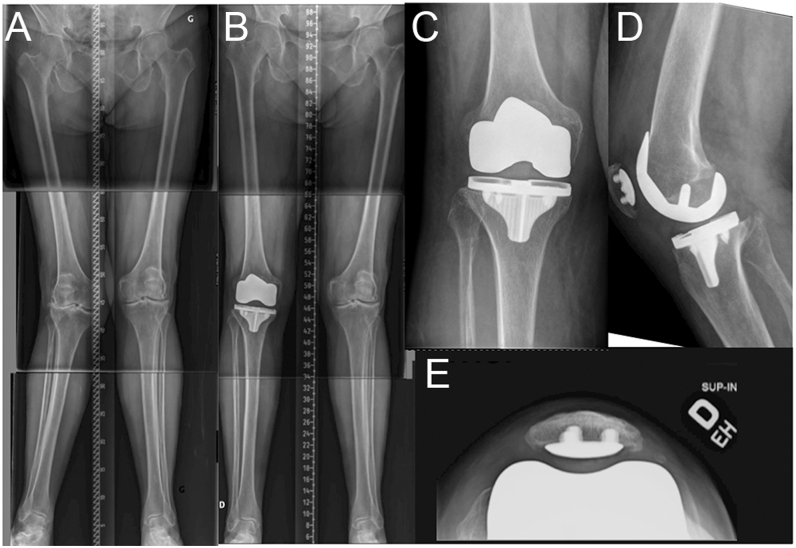
Mild valgus case. (A) Preoperative long-leg radiographs of an 82-year-old female with a mechanical HKA of 17° valgus and an arithmetic HKA of 6° valgus (distal femur 9° valgus and proximal tibia 3° varus). (B, C, D, E) Postoperative radiographs after an uncemented rKA-TKA. According to the rKA protocol, the femur valgus was reduced to 5°, while the tibia was maintained at 3° varus, resulting in a HKA of 2° valgus. The femoral implant was placed without femoral rotation, and the patella was resurfaced. Limited pie-crusting of the posterolateral structures was necessary to obtain ligament balance.

After removing significant osteophytes and evaluating the wear status of the joint surfaces, since cartilage and bone loss can affect the gaps, a careful assessment of joint laxity should be performed. The joint can then be classified as follows:

- Normal laxity (most) (Fig. 5).
- Generalized hyperlaxity (often).
- Lateral contracture (rarely).
- Medial laxity (elongated MCL, rarely).

Each of these situations requires a different strategy. Regardless of the scenario, we recommend aiming for a tight and balanced medial compartment, with 1–2 mm of gap opening at 10 degrees of flexion and 2–3 mm at 90 degrees of flexion. To perform the gap assessment precisely, advanced precision tools such as robotic systems can provide objective and reproducible measurements.

In patients with generalized hyperlaxity (‘floppy’ knees), we tighten the joint by undercutting the tibia by 2–5 mm, depending on the degree of laxity that needs correction. We do not recommend undercutting the distal or posterior femur, as this may alter the femoral flexion axis, particularly in cases involving a fixed flexion contracture.

For cases with mild MCL laxity (up to 3–4 mm), we undercut the tibia by 2–3 mm, using the naturally greater lateral laxity to balance the joint. This approach ensures symmetric gaps rather than restoring the patient’s native laxities. This strategy also helps reducing patellar height in presence of patella alta.

For MCL elongation greater than 3–6 mm, lateral soft tissue releases or a constrained posterior-stabilized insert is necessary, while a hinged implant is indicated in cases of an incompetent MCL.

In cases with lateral soft tissue contractures, any undercutting of the lateral compartment (femur or tibia) should be avoided, as this contradicts the fifth principle of rKA, which emphasizes performing anatomical corrections on the worn side (41). In these cases, soft tissue releases should be performed on the affected structures while carefully considering the potential impact on the peroneal nerve. Lateral contractures are most commonly associated with a fixed flexion deformity greater than 20 degrees and lateral retinacular contracture. In such cases, a lateral surgical approach may be beneficial. In the authors’ opinion, significant attention has been given to contracture of the LCL; however, other structures, such as the popliteal tendon, the lateral capsule, and the iliotibial band, might play an even more significant role. If only the LCL is addressed and/or released, these additional contractures could impact outcomes. When applying rKA boundaries and increasing the tibial varus orientation, before performing ligamentous release, we will accept the reduction of the lateral flexion gap, thereby modifying the trapezoidal flexion space toward a more rectangular shape. We will perform lateral release if the space becomes inverted (medial flexion gap larger than the lateral gap).

When using a calipered technique, computer navigation, or robotic assistance, joint surface landmarks should be referenced from the most reliable areas, with intact cartilage being the ideal reference. In cases of severe bone loss, assessing ligament laxity can help determine the native joint line level (20). Those landmarks are important because the objective is to restore the knee to its pre-arthritic state. It is important to mention that lateral femoral condylar bone loss, which can be present in valgus knees, may exaggerate the measured distal femoral valgus angle on radiographs and should be considered when interpreting coronal alignment.

### Implant and alignment

As we believe that some valgus knee anatomies should be considered pathological and not reproduced, we use the rKA proposed by Vendittoli. rKA limits the femoral valgus to 5° valgus and the arithmetic HKA to 3° valgus (41). These limitations are crucial to prevent the replication of ‘deviant’ anatomies and to accommodate the constraints of current femoral implant designs (21). Here again, precision tools such as navigation or robotics are valuable for monitoring intraoperative alignment and ensuring component placement remains within an acceptable range. For most valgus knees where the native patellofemoral joint is congruent on the skyline view, the standard rKA protocol should be applied. Femoral axial alignment should remain neutral, ensuring symmetric resection of the posterior condyles.

In MA TKA, femoral external rotation was originally performed to balance the flexion gap by compensating for the neutralization of the tibial anatomical varus. However, in valgus knees, where the tibial mechanical axis is often neutral or in valgus, adding external rotation can create a flexion imbalance (6, 14). This fundamental flaw in systematic MA alignment should be avoided in varus and valgus knees for several reasons (3). First, altering the femoral component’s rotational alignment disrupts the patient’s native anatomy, affecting the femoral flexion axis and altering tibial and patellofemoral kinematics (6, 57). Second, the relationship between anatomical landmarks varies significantly among individuals, making standardized adjustments unreliable (42). Third, it creates mediolateral flexion gap imbalances, height asymmetry, lateral prosthetic overhang, and medial undercoverage (6, 58). In addition, some osteoarthritic valgus knees exhibit posterolateral wear. In these cases, the wear should be accounted for when positioning the posterior condylar reference cutting guide, once again to respect the pre-arthritic knee state. Furthermore, we do not perform routine patellar resurfacing. This approach is adequate for most cases, including those with atypical anatomies.

**Figure 6:**
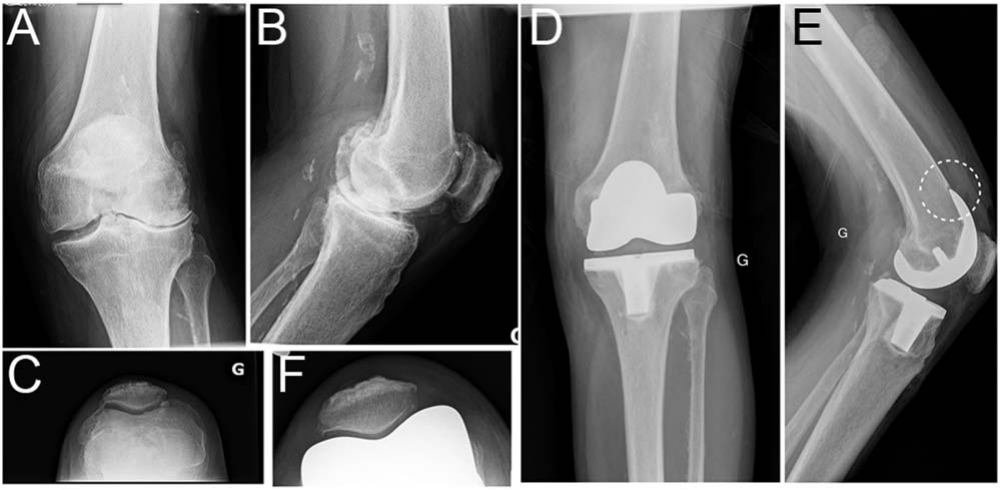
Trochlea dysplasia. (A, B, C) Preoperative long-leg radiographs of an 86-year-old male with a moderate valgus left knee. The knee was presenting a shallow and dysplastic trochlea. (D, E, F) During surgery, femoral size was between size 6 and 7. We selected the smaller size with a posterior reference and voluntarily accepted to notch the anterior cortex by 2 mm to avoid overstuffing the dysplastic patellofemoral joint (dashed circle). Following the rKA protocol, we resurfaced the medial femoral condyle (implant thickness from zones of intact cartilage) and removed 7 mm from the subchondral bone on the lateral compartment to reduce the femoral valgus from 6 to 5°. Tibial cut angulation was 3° for an arithmetic HKA of 2° valgus. The knee was stable, and a medially constrained CR insert was selected.

In cases where there is a pathological patellofemoral joint (presence of a patellar tilt or subluxation preop), to enhance patellofemoral stability and function, additional surgical steps may be considered in some cases (20):

- Downsizing, accepting 1–2 mm of notching in cases with a dysplastic or shallow trochlea (Fig. 6).
- Lateralizing the implant to the lateral condylar edge (Fig. 6).
- Using a femoral implant with a large trochlea and low lateral ridge.
- Patella resurfacing, medializing the button combined with lateral facetectomy.

If a specific pathology is present, such as patella alta, severe lateral retinacular contracture, or a malunited patellar fracture, it may need to be addressed separately. In such cases, the authors recommend using:

- Lateral arthrotomy (Keblish approach (59)) combined with Z-plasty of the retinaculum, which further reduces lateral retinacular tension.
- Tibial tuberosity osteotomy (especially in cases with pathologic TA-GT or patella alta).

These strategies are rarely performed but can enhance patellofemoral stability and help address the limitations of MA and KA techniques in patients with complex patellar pathology.

## The limits of personalized TKA for valgus knee

In the authors' opinion, alignment boundaries should continue to be used, as insufficient evidence supports the superiority or safety of unrestricted KA in all patients (21). Furthermore, we believe certain extreme deformities are likely pathological and should not be replicated (11). In mild valgus cases, the primary contributor to malalignment is the femur, whereas in extreme cases, both the femur and tibia contribute to the valgus alignment. In addition, in 17% of cases, the distal femoral joint surface is oriented at more than 5° of valgus, necessitating a modification of the native anatomy with rKA (41). The author believes preserving femoral anatomy is fundamental to maintaining proper knee kinematics. Therefore, in severe valgus knees, after reducing the femoral valgus to 5°, any remaining adjustment is performed on the tibia to obtain an arithmetic HKA of 3° (41).

**Figure 7:**
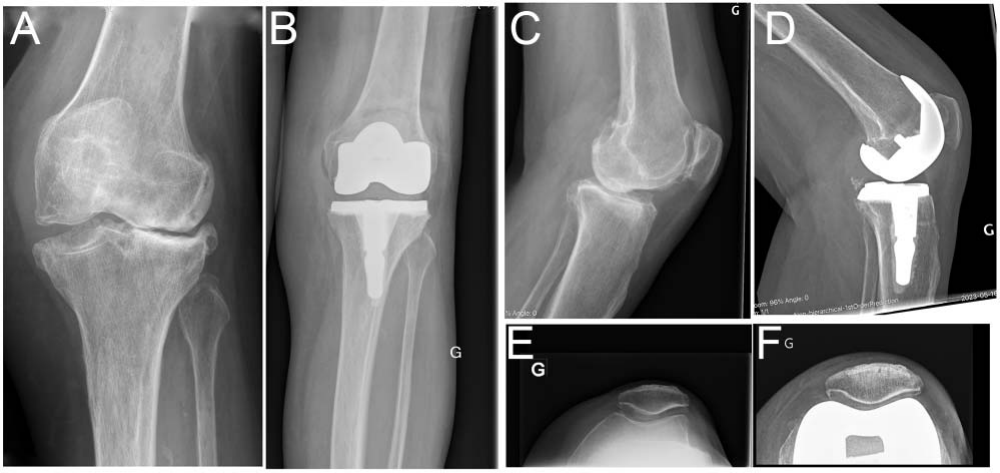
Valgus with global instability. (A and C) Preoperative radiographs of an 80-year-old female with a left knee in severe valgus (mechanical HKA of 9° valgus). The knee was grossly loose both medially and laterally upon clinical examination but had a firm end feel. (B and D) Using the rKA protocol, we resurfaced the medial femoral condyle (implant thickness from zones of intact cartilage) and removed 5 mm from the subchondral bone on the lateral compartment to reduce the femoral valgus from 7 to 5°. To compensate for the soft tissue hyperlaxity and minimize the polyethylene thickness, we under-resected by 3 mm the tibial side (7 mm from zones of intact cartilage for a 10 mm implant). Tibial cut angulation was 3° for an arithmetic HKA of 2° valgus. MCL laxity was estimated to be 4–5 mm at 10° of flexion, and a semi-constrained insert was used in combination with a short, cemented stem.

**Figure 8:**
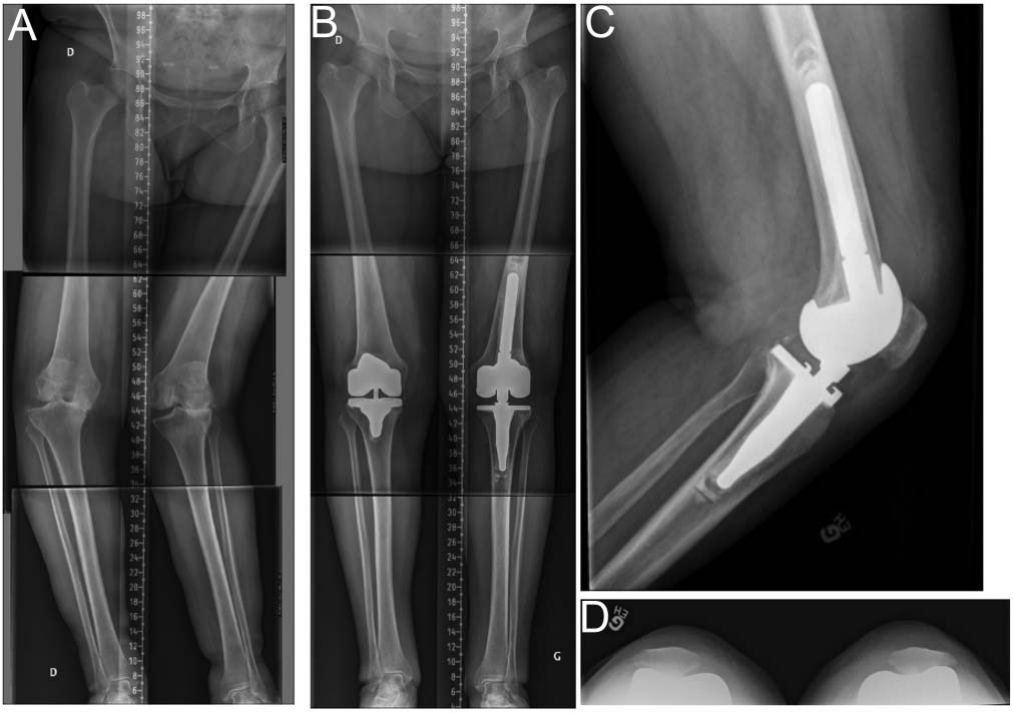
Severe windswept lower limbs. (A) Preoperative long-leg radiographs of a 78-year-old female with a right knee in severe varus (mechanical HKA of 21° degrees) and a left knee in severe valgus (mechanical HKA of 28° valgus). (B, C, D) Using the rKA protocol, the right knee was left with an imbalance of 4 mm after MCL release and required a semi-constrained insert. On the left side, the MCL was incompetent, and a hinge TKA was implanted.

Correcting outlier anatomy inevitably creates imbalances. Imbalances greater than 3 mm typically require soft tissue releases. Another way to express this is that soft tissue releases are generally necessary when modifying the native anatomy by 2–3°, which occurs in approximately 10–20% of cases (6). When an imbalance greater than 5 mm is created or is due to MCL elongation/insufficiency, the use of constrained implants should be considered. We suggest using a semi-constrained bearing for residual imbalances of 2–3 mm after ligamentous release (Fig. 7). In cases of severe MCL insufficiency, a hinged implant is the preferred choice (Fig. 8).

## Outcomes and patient satisfaction of personalized arthroplasty performed in valgus knees

Accumulating data demonstrates favorable early and long-term outcomes of personalized arthroplasty in patients with varus and valgus knees (4, 60, 61, 62). Promising early results in a cohort of 26 KA-TKAs performed in valgus cases with an anatomical femorotibial angle of >10 degrees (11–23°) have been published (27). With mid-term outcomes, Howell *et al.* (61), in a cohort of 198 patients with both varus and valgus knees who underwent KA-TKA, reported at an average follow-up of 4 years a reoperation rate of 1.5% (3/198). All three revisions were performed in patients with more extreme valgus phenotypes due to patellofemoral symptoms. Although the author did not explain the study’s revision rate clearly, implant design and extreme anatomy replication may have played a role. A recent study including 298 valgus knees demonstrated that superior functional and satisfaction outcomes were achieved when severe valgus anatomies (>10°) were left in residual valgus (4–9°) (63). The authors attributed the higher satisfaction to the fewer soft tissue releases required, reducing the risk of medial instability. Another recent study with 11.3 years of follow-up reported implant survivorship of 99% in a cohort of cemented rKA-TKA in both varus and valgus knees (4), while the unique revision occurred in a valgus knee and was linked to an external femoral malrotation (surgical error).

In the article published by Howell *et al.* on 222 KA-TKAs with 16 years of follow-up, five of the twelve (42%) complications were related to patellofemoral joint problems (three patellar instability and two loose patellar implants) (62). However, to the best of our understanding, while all instabilities occurred in patients with valgus HKA (1–3 degrees), the loosening of implants was observed exclusively in varus-aligned TKA. Finally, Dosset *et al.* reported excellent outcomes of KA-TKA in a cohort of valgus and varus knees at a mean follow-up of 13 years, with results comparable to MA TKA, including similar rates of reoperations (survivorship: 82% for the KA group vs 84% for the MA group (*P* = 0.83)), complications, and PROMs (60). Interestingly, patellar complications were reported as the main cause of reoperation (11.0%) (60). In contrast, no patellar issues were reported in the long-term rKA-TKA study by Morcos *et al.* (4). In the authors’ experience with rKA over 14 years, no cases of patellofemoral joint instability requiring revision have been observed. Thus, setting boundaries for HKA might help prevent this complication, as the current implants may not be compatible with some deviant anatomies (21, 64).

Regarding the satisfaction rate, for some authors, a postoperative valgus alignment of more than 5 degrees may be cosmetically less desirable to patients (5). While we agree that not every anatomy should be reproduced and believe in respecting predefined boundaries, cosmetic concerns should be considered secondary (21). While some patients might prefer a straight leg aesthetically, it can be assumed that most, particularly in the older population, would prioritize function over aesthetics when the reasons for maintaining a valgus alignment postoperatively are adequately explained. This was demonstrated in a study by Morcos *et al.*, which showed that 95% of patients in a cohort of both varus and valgus knees were satisfied or very satisfied with their rKA-TKA surgery, with a minimum follow-up of 10 years (4). Another article showed that the proportion of satisfied or very satisfied patients did not differ significantly between those who underwent KA-TKA with valgus >10° and those with valgus ≤10°, with rates of 88 and 93%, respectively (27).

That being said, other factors should be considered. First, was the patient satisfied with their limb alignment before developing end-stage osteoarthritis? Second, the overall patient’s morphology must be taken into consideration. For instance, in morbidly obese patients, reproducing a valgus limb alignment might be undesirable due to increased friction between the adipose tissues of the legs, leading to gait discomfort. Finally, as mentioned earlier, adjacent joint pathologies in the hip or foot, or a varus alignment of the contralateral leg (windswept deformity), may necessitate valgus knee correction.

## Conclusion

As time progresses, advancements in modern implants, combined with a deeper understanding of knee anatomy and biomechanics, have led to higher satisfaction rates in TKA. We believe valgus knees benefit just as much as varus knees from a personalized surgical approach. However, each knee should be carefully evaluated individually, considering multiple specific variables to achieve optimal outcomes.

## ICMJE Statement of Interest

The authors declare that there is no conflict of interest that could be perceived as prejudicing the impartiality of the research reported.

## Funding Statement

The Maisonneuve-Rosemont Foundation funded this research, supporting the arthroplasty fellowship program.

## Author contribution statement

GB and P-AV were responsible for drafting and critically revising the manuscript. M-OK and VM were responsible for critically revising the manuscript.
